# PLA2G2A is a candidate biomarker for sepsis diagnosis in mice through machine learning algorithms

**DOI:** 10.3389/fimmu.2026.1937718

**Published:** 2026-09-30

**Authors:** Hong Zhu, Pai Peng, Shiquan Han, Rongli Yang

**Affiliations:** 1 Graduate School of Dalian Medical University, Dalian, Liaoning, China; 2 Department of Critical Care Medicine, Central Hospital of Dalian University of Technology, Dalian, Liaoning, China; 3 Department of Peripheral Vascular Intervention, Central Hospital of Dalian University of Technology, Dalian, Liaoning, China; 4 Faculty of Medicine, Dalian University of Technology, Dalian, Liaoning, China

**Keywords:** bioinfomatics, biomarkers, machine learning, secreted phospholipase A2 (PLA2G2A), sepsis

## Abstract

**Objective:**

In this study, machine-learning screening was implemented on human transcriptomic data from public GEO datasets and independent experiments were served to validate candidate biomarkers for sepsis in mice models.

**Methods:**

Microarray data were obtained from the GSE236713 dataset, and differentially expressed genes (DEGs) were screened. We carried out enrichment analysis with the help of gene ontology (GO) and Kyoto encyclopedia of genes and genomes (KEGG). We utilized machine learning algorithms, such as least absolute shrinkage and selection operator (LASSO), logistic regression (LR), support vector machine (SVM). For the key genes, we conducted single-gene enrichment and immune Infiltration Analysis. We selected C57BL/6J SPF-grade male mice to construct a sepsis model. The proteins were then detected via western blotting (WB), tissue homogenization, tissue staining, immunohistochemistry (IHC), and cytokines.

**Results:**

Multiple groups of DEGs were identified via dataset analysis. Weighted gene co-expression network analysis (WGCNA) was used to identify related modules. Machine learning was used to screen key genes, such as cholinergic receptor muscarinic 1 (CHRM1) and Secreted phospholipase A2 (sPLA2-IIA) (encoded by *PLA2G2A*). sPLA2-IIA expression was also higher in the mouse sepsis model group than the sham group. And its expression was implying regulatory effects. The successful mouse model is characterized by elevated levels of IL-6, TNF-α, and multi-organ damage.

**Conclusion:**

In this study, we identified PLA2G2A as the candidate biomarker for diagnosis of sepsis in mice models through machine learning algorithms. And the data obtained may be helpful for subsequent research on sepsis pathogenesis.

## 1 Introduction

As an abnormal immune response to infection, sepsis can trigger the systemic inflammatory response syndrome and the mortality rate of sepsis exceeds 30% (1). Pathogen infection is a major contributor to the high mortality rate associated with sepsis. The development of an abnormal immune response can range from excessive inflammation to immunosuppression. In this process, sepsis-induced abnormalities are manifested by a significant reduction in the number of immune cells and dysfunction in immune function (2–4).

Clinicians currently rely on some immune biomarkers to diagnose and treat sepsis, such as procalcitonin, lymphocyte counts, and ferritin (5–7). However, these markers somehow lack specificity and cannot fully satisfy the accurate clinical diagnosis and effective treatment of sepsis. Bioinformatics analysis can be used to screen out genes from a large amount of data. The data is closely related to the phenotype with the support of high-throughput sequencing technology. Protein data were transformed into biologically meaningful information through GO functional enrichment and KEGG pathway enrichment analyses. The application of machine-learning algorithms enhances the accuracy and efficiency of screening key genes for the development of sepsis. By comprehensively analyzing the feature importance scores of different algorithms, genes are screened as the key genes for sepsis with high importance scores in multiple algorithms. This method avoids the interference of human factors and improves the reliability and objectivity of the screening results.

The LASSO algorithm achieves variable screening and model simplicity through contraction and selection of coefficients. When dealing with high-dimensional data, the LASSO can not only identify genes most relevant to the development of sepsis but also avoid overfitting effectively. LR models have been proven to perform equally well. They tend to be more robust against data imbalances, less biased, computationally more efficient, and have better explainablity compared to other machine learning algorithms (8, 9). The support vector machine (SVM) algorithm realizes the effective classification of data by finding the optimal classification hyperplane. The gaussian mixture model (GMM) algorithm classified the data by modeling the probability distribution. It addresses the uncertainty and complexity of the data.

Secreted phospholipase A2 (sPLA2-IIA) has been proven to participate in lipid metabolism and inflammatory responses, as an important member of the secreted phospholipase A2 family (10). In the lipid metabolism pathway, it exerts a critical influence on the composition and distribution of lipids by catalyzing the hydrolysis of specific phospholipids. sPLA2-IIA may participate in inflammatory mediator responses. This enzyme primarily targets extracellular phospholipids and has implications in host antimicrobial defense, inflammatory response, and tissue regeneration (11). *PLA2G2A* has also been implicated in the recruitment of immune cells (12). Previous clinical studies have reported altered circulating sPLA2-IIA (encoded by *PLA2G2A*) concentrations in patients with severe sepsis and septic shock, and its diagnostic potential for sepsis has been preliminarily assessed in clinical cohorts (13–15). Nevertheless, few studies have further explored its pathological function and downstream immune-cell-centred mechanisms during sepsis.

In the present work, we applied integrated machine-learning and network-analysis on human transcriptomic data from sepsis with diverse infection sources to prioritise *PLA2G2A*. We further characterised its in-vivo expression profile in the CLP mouse model and confirmed its correlation with pro-inflammatory cytokines, providing additional mechanistic evidence for *PLA2G2A* in sepsis pathogenesis.

## 2 Materials and methods

### 2.1 Animals

6-week-old C57BL/6J SPF-grade male wild-type mice were purchased from Huafukang Biotechnology Co. Ltd (Beijing, China). Genetic substrain authentication was performed by Suzhou Xishan Biotechnology Co., Ltd. (Suzhou, China) using a SNP-based genotyping panel (G1001+G1002) against the Jackson Laboratory reference database. The selection of male mice was driven by their genetic consistency, well-characterized biology, hormonal stability, and practical advantages in experimental design. The mice were fed in the SPF-grade laboratory at Shouke Lingfu Biotechnology Co. Ltd (Beijing, China). The temperature was set at 22 ± 1 °C, the relative humidity was 40%-60%, and the light-dark cycle was set as 12 h: 12 h.

### 2.2 Commercial reagents and antibodies

All commercial reagents and antibodies were used according to the manufacturers’ instructions unless otherwise specified. The sources and detailed information of key reagents and antibodies are summarized in **Table 1**. Of note, the sPLA2-IIA antibody and ELISA reagents used in this study may recognize truncated mutant *PLA2G2A* peptides rather than enzymatically active wild-type protein, given the *PLA2G2A* frameshift mutation carried by C57BL/6J mice.

**Table 1:** Inclusion of dataset information.

ID	Year	Platform	Samples number	Country
GSE236713	2024	GPL17077	Healthy(30)	United Kingdom
			Pulmonary(200)	
			Abdominal(124)	

### 2.3 Bioinformatics analysis

Gene expression profiles and clinical data of patients with sepsis were downloaded from the Gene Expression Omnibus (GEO) database. Filtering conditions including keywords, species, and sample types were set, followed by data sorting and conversion into a matrix format. The data were normalized, depolarized, batch effect-removed, and missing values were deleted. The “limma” package was used to calibrate the gene expression matrix and match gene names. The dataset was screened for differentially expressed genes (DEGs) and stratified into three groups: healthy, sepsis (lung infection), and sepsis (abdominal infection).

Differential analysis was performed using a generalized linear model with R software and the “limma” package, with screening criteria set. Volcano plots and heatmaps were generated for visualization. Gene Ontology (GO) and Kyoto Encyclopedia of Genes and Genomes (KEGG) enrichment analyses were conducted on co-expressed DEGs. Weighted Gene Co-Expression Network Analysis (WGCNA) was applied to identify relevant modules, followed by construction of a gene co-expression network and screening of Hub genes. Machine learning algorithms were utilized for diagnosis and screening of co-expressed genes. Additionally, single-sample gene set enrichment analysis and immune infiltration analysis were performed to analyze the co-expressed genes.

Machine-learning algorithms were implemented in R. LASSO-penalised logistic regression was utilised for feature-selection to filter candidate genes from the intersection of DEGs and WGCNA hub-genes. Logistic regression, support vector machine (SVM), random forest (RF), Gaussian mixture model (GMM), LDA and QDA served only as exploratory comparative classifiers to rank gene-set discriminatory potential. It should be noted that these models were used solely for candidate gene screening for subsequent wet-lab validation, and are not intended as clinical diagnostic tools. Fixed random seed set.seed =12345 was used for reproducibility, with 5-fold cross-validation repeated three times; no separate hold-out test set was generated. Within each cross-validation iteration, normalisation and batch-effect correction were performed exclusively on training-fold data, and fitted parameters were applied to corresponding validation fold. LASSO feature-selection was nested within each training fold, rather than computed on the full dataset ahead of cross-validation. Confusion matrices count individual samples rather than unique patients; GSE236713 contains multiple longitudinal samples per patient.

### 2.4 Construction of mouse sepsis model

Sepsis models were constructed by cecum ligation and perforation (CLP). Under 4% isoflurane (#R510-22-10, China) anesthesia, a midline laparotomy was performed. And then the cecum was ligated 1–2 cm from its end using 4/0 silk sutures (E-641G, Ethicon, USA). The cecum was perforated by 18-gauge needle from the mesentery towards the antimesenteric direction. Fecal material was gently expressed through the perforations. The cecum was returned and closed the abdomen with absorbable sutures. Sham-operated mice underwent the same procedure without ligation or perforation. Postoperatively, mice can be received subcutaneous 1 ml 0.9% NaCl (#G4702, China). Mice were sacrificed at 6 h, 12 h, 24 h, 48 h and 72 h post-CLP or sham surgery for tissue collection; plasma was harvested at 24 h, 48 h and 72 h for ELISA measurement. All experimental time-points used independent terminal-sacrificed animals; serial repeated blood sampling was not performed on individual mice.

### 2.5 Tissue homogenate preparation and proteins extraction

Grinding beads (3 mm) (#G0103-200G, Servicebio, China) were placed into marked tubes. Every tube was added 4 pieces of beads. The tissue was weighed and cut it into small pieces. Tissue pieces were rinsed 5 times with phosphate buffered saline (#C0221A, China) and absorbed excess liquid. Based on the tissue weight, pre-chilled RIPA lysis buffer (#P0013B, China) was added at a ratio of 5 μl of lysis buffer per 1 mg of tissue. The tissue was ground using a TissueMaster™ High-Throughput Tissue Homogenizer (E6618, China) for 60 sec per cycle, repeated 3 times. The tissue homogenates were transferred to Eppendorf tubes. The tubes were shaken for 30 sec every 10 min and incubated on ice for 30 min to facilitate complete tissue lysis. The samples were centrifuged at 4 °C and 12,000 rpm for 25 min to separate proteins. The supernatants were promptly aspirated using a pipette and immediately transferred to new labeled centrifuge tubes.

### 2.6 Cytokine enzyme-linked immunosorbent assay

To determine the levels of sPLA2-IIA (#EK1945, China, detection range: 31.2pg/ml-2000pg/ml), Interleukin 6 (IL 6) (#EK0411, China, detection range: 15.6 pg/ml-1000 pg/ml) and Tumor Necrosis Factor-Alpha (TNF-α) (#EK0527, China, detection range: 15.6 pg/ml-1000 pg/ml). The blood samples were collected in EDTA tubes (#367841, USA) on the Day 1, Day 2, and Day 3 after modeling and stayed at 37 °C for 1–2 h. After lysis, samples were centrifuged at 4 °C and 3,000 rpm for 10 min. ELISA kits were utilized following the manufacturer’s protocols. The values were red at 450 nm for each well with a Bio-Tek Synergy HTX microplate reader (Bio-Tek, USA).

### 2.7 Paraffin sectioning technique

Tissues were fixed with 10% formalin (#F5554, USA) (fixative volume 5–10 times the tissue volume) at room temperature for 24–48 hours, then trimmed to appropriate size, placed in embedding cassettes, and processed for paraffin embedding (total 16 hours). Paraffin blocks were trimmed and sectioned at 5 µm; paraffin ribbons were placed in a 40-45 °C water bath for mounting onto slides, air-dried for 30 mins, and baked in a 45-50 °C oven overnight.

### 2.8 Hematoxylin-eosin staining

Before a series of graded ethanol dehydration, The above paraffin sections were paraffinized and fixed in 4% paraformaldehyde (#P0099, China). The samples were subsequently soaked in distilled water for 2 min. Hematoxylin staining solution (#C0107, China) was dropped on the sections for 5 min of staining, then rinsed with tap water.

### 2.9 Immunohistochemistry

Tissue sections were sequentially dewaxed in xylene, rehydrated in 95% ethanol, and rinsed with distilled water. Samples was quenched by incubation in 3% hydrogen peroxide in methanol in the dark. Antigen retrieval was performed by heating sections in Tris-EDTA antigen retrieval buffer (P0084, China) in a microwave, followed by natural cooling and repeated washing with phosphate-buffered saline (PBS). Sections were blocked with rabbit serum (#AR1010, China) for 30 min at 37 °C. After removing the blocking solution, sections were incubated with rabbit polyclonal anti-PLA2G2A antibody (#DF6366, China) diluted 1:100 overnight at 4 °C. Probed with biotin-conjugated goat anti-rabbit IgG (H+L) secondary antibody #(SA00004-2, China) diluted 1:500. Color development was performed using diaminobenzidine chromogenic solution (#D12384, USA). Stained sections were examined under a light microscope, and average optical density values were quantified using Image-Pro Plus 6.0 software.

### 2.10 Western blot

The samples were mixed with loading buffer (#26616, USA) and then denatured by heating at 100 °C for 5 min. According to the BCA assay results, we loaded wells with 30 μg target protein and 5-10 μg reference protein. The 12.5% one-step color polyacrylamide gel electrophoresis was conducted by using Omni-Easy™ Gel Rapid Preparation Kit (PG212, China). Then the gel were transferred to the polyvinylidene fluoride (PVDF) membranes. After blocking, membranes were incubated with primary antibodies: anti-PLA2G2A (Affinity, #DF6366, China) at 1:1000 dilution, and β-actin monoclonal antibody (Proteintech, #66009-1-Ig, China) at 1:5000 dilution. Subsequently, membranes were probed with HRP-linked anti-rabbit IgG secondary antibody (Cell Signaling Technology, #7074, USA) at 1:3000 dilution. The following antibodies used are listed in **Table 1**.

### 2.11 Quantitative real-time PCR

Total RNA was isolated from lung, kidney, liver tissues and peripheral blood cells using RNA extraction solution (Servicebio, Cat. G3013) with chloroform substitute (Servicebio, Cat. G3014) following the manufacturer-described procedures. RNA purity and concentration were determined by NanoDrop 2000 spectrophotometer. cDNA was synthesized from total RNA with SweScript All-in-One RT SuperMix for qPCR (Servicebio, Cat. G3337) in a 20 μL system under the program of 25 °C for 5 min, 42 °C for 30 min and 85 °C for 5 sec. Quantitative real-time PCR was performed on a Bio-Rad CFX Connect system using 2×Universal Blue SYBR Green qPCR Master Mix (Servicebio, Cat. G3326). The primer sequences for PLA2G2A and reference gene Gapdh are listed in **Supplementary Table S2**. Relative gene expression was quantified by the 2^-ΔΔCt^ method. All qPCR reactions were run in technical triplicates with at least three independent biological replicates per group.

### 2.12 Statistical analysis

Data were presented as Mean ± SEM (ELISA measurements were performed with n = 4 biological replicates, histology analyses were conducted using n = 6 biological replicates and qPCR were implement with n = 4 biological replicates). To assess differences, one-way ANOVA (Kruskal–Wallis test) test for multiple comparisons was used with GraphPad Prism version 9.4.1 (USA).

## 3 Results

### 3.1 GSE236713 data download and processing

In this study, the GSE236713 dataset is selected via the GEO database in strict accordance with **Figure 1**. The key screening conditions are as follows: “Keyword” is set as “sepsis”; “Species” is “*Homo sapiens*”; “Sample type” is designated “whole blood/ribonucleic acid”; and the study population is defined as human. The patients are classified into three groups: healthy (n=30), pulmonary infection samples (n=200), and abdominal infection samples (n=124) (**Table 1**).

**Figure 1:**
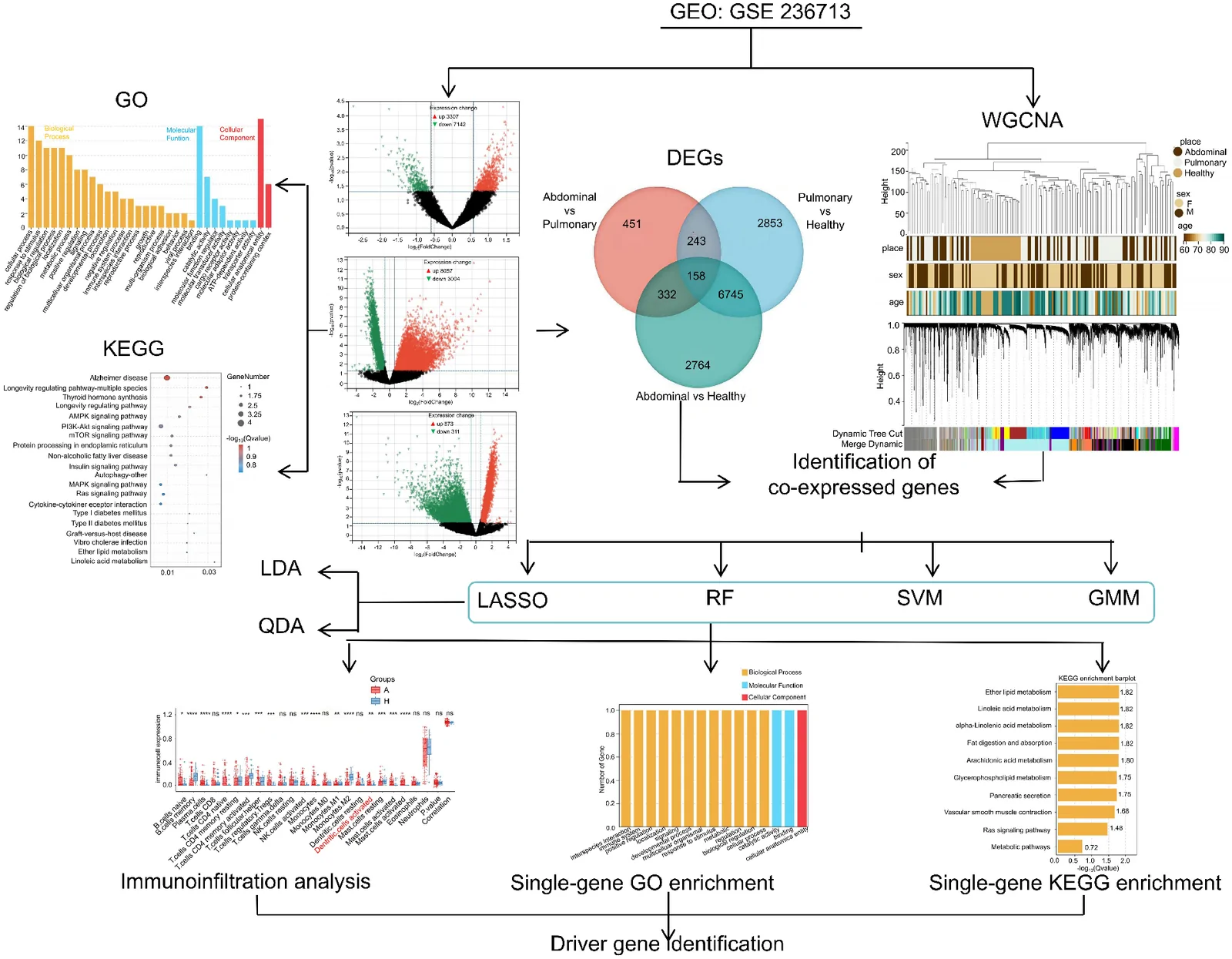
Flow chart of key gene screening based on the GSE236713 dataset combined with machine learning algorithms. Raw transcriptomic data were downloaded from GEO database (GSE236713). Differential expression analysis was performed to identify DEGs, while weighted gene co-expression network analysis (WGCNA) was applied to detect co-expressed gene modules. Candidate gene sets derived from DEGs and WGCNA hub genes were subjected to subsequent machine-learning pipelines. LASSO-penalised logistic regression was used for feature selection, followed by comparative exploratory classifiers including random forest (RF), support vector machine (SVM), Gaussian mixture model (GMM), linear discriminant analysis (LDA), and quadratic discriminant analysis (QDA). Machine-learning was applied solely for exploratory candidate gene screening. Downstream analyses contained immune-infiltration analysis, single-gene GO and KEGG enrichment, for the final identification of potential driver genes.

### 3.2 Differential expression and functional enrichment analysis based on the GSE236713 dataset

Differentially expressed genes (DEGs) were screened via the R package limma with the threshold of log_2_(FC) ≥ 1.5 and p < 0.05 to compare transcriptional profiles across healthy, pulmonary sepsis and abdominal sepsis cohorts. Between healthy and pulmonary sepsis groups, 10,449 DEGs were identified, including 3,307 upregulated and 7,142 downregulated genes (**Figure 2A**). A total of 11,061 DEGs were detected when comparing healthy and abdominal sepsis samples, consisting of 8,057 upregulated and 3,004 downregulated transcripts (**Figure 2B**). In the pairwise comparison between pulmonary and abdominal sepsis groups, 1,184 DEGs were obtained 873 upregulated, 311 downregulated (**Figure 2C**). Volcano plots (**Figure 2D**) illustrated distinct expression patterns across subgroups, with upregulated genes dominating sepsis-related transcriptional alterations, and Venn analysis identified 158 overlapping DEGs shared by all three comparison sets. To decipher the biological roles of these shared DEGs, GO and KEGG enrichment analyses were further conducted. GO enrichment uncovered 22 biological process terms and 8 molecular function terms (**Figure 2E**): prominent biological processes covered cellular activities, stimulus response, biological regulation, localization, metabolism, signal transduction and immune responses; major molecular functions included molecular binding, catalytic activity, regulatory function and ATP-dependent activities, while cellular component terms were mainly enriched in cell structures and protein complexes. KEGG pathway enrichment (**Figure 2F**) highlighted critical cascades including the AMPK, PI3K-Akt, mTOR, insulin signaling pathways, endoplasmic reticulum protein processing and multiple autophagy-related pathways.

**Figure 2:**
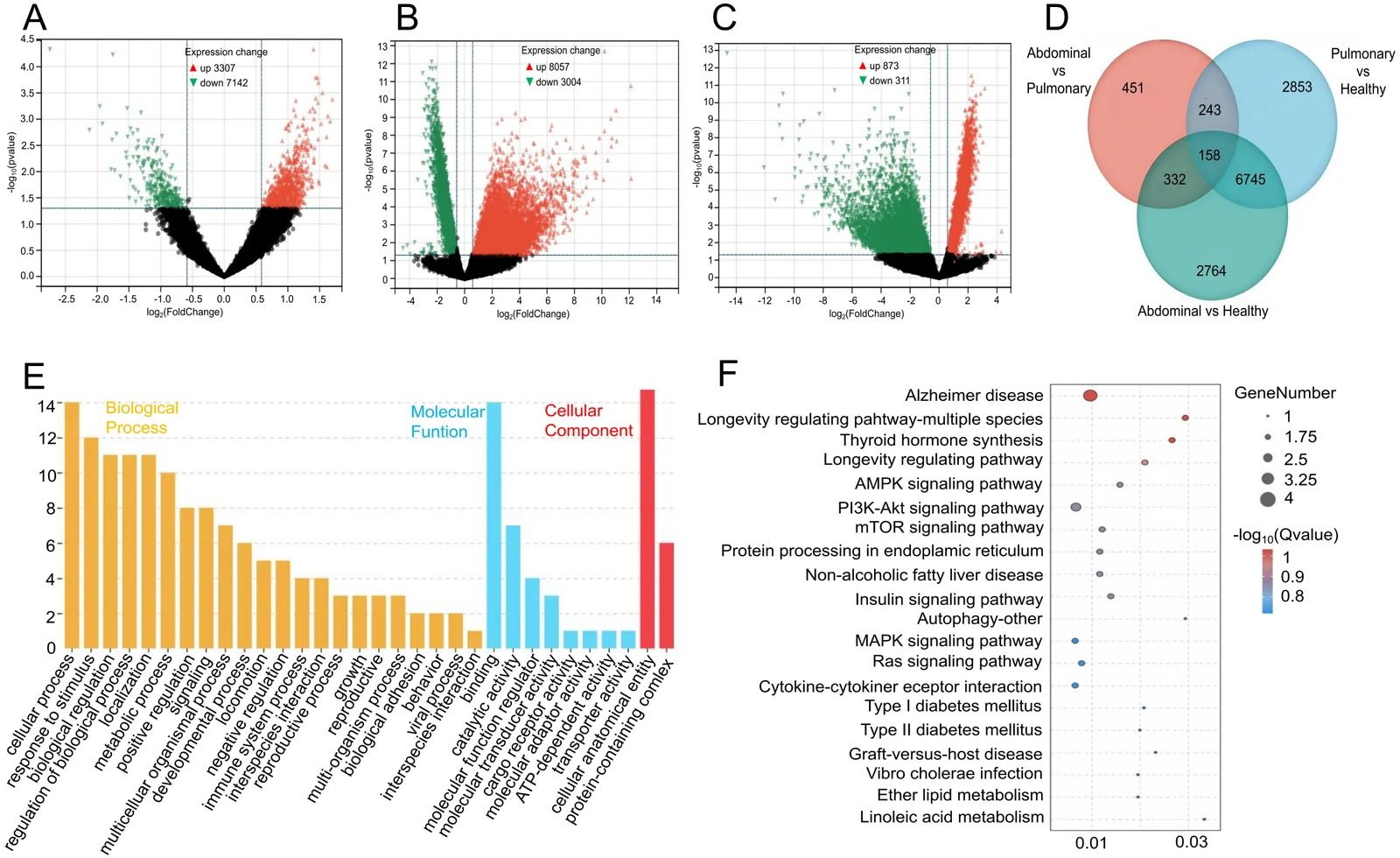
Identification and functional enrichment of DEGs in sepsis derived from GSE236713 dataset. **(A)** Volcano plot illustrating DEGs in pulmonary sepsis versus healthy controls. A total of 3,307 genes were significantly upregulated and 7,142 genes downregulated. **(B)** Volcano plot of DEGs in abdominal sepsis versus healthy controls, with 8,057 upregulated and 3,004 downregulated genes identified. **(C)** Volcano plot showing DEGs between pulmonary sepsis and abdominal sepsis groups, including 873 upregulated and 311 downregulated genes. **(D)** Venn diagram displaying overlapping DEGs across healthy, pulmonary sepsis, and abdominal sepsis cohorts; 158 shared DEGs were screened out. **(E)** GO enrichment analysis categorizing all DEGs into biological process, cellular component, and molecular function terms. **(F)** Bubble plot for KEGG pathway enrichment of shared DEGs. Bubble size corresponds to the number of enriched genes per pathway, while bubble color reflects enrichment significance (−log_10_(Q value)). DEGs, Differentially Expressed Genes; GO, Gene Ontology; KEGG, Kyoto Encyclopedia of Genes and Genomes.

### 3.3 Weighted gene co-expression network analysis and hub gene screening

Clinical covariates including sample grouping (healthy, pulmonary and abdominal sepsis), sex and age were visualized, with patient ages predominantly concentrated from 60 to 90 years, as shown in **Figure 3A**. After MAD filtering, an expression matrix comprising 6,766 genes and 97 samples was retained to remove noisy transcripts. Sample clustering identified no outlier samples. The soft threshold power β = 14 was selected, at which the scale-free topology fitting R² reached 0.86 and the average connectivity was 9.03, as detailed in **Figures 3B, C**. The clustering height profile exhibited cut-off values for dynamic tree cutting and module merging, supporting subsequent gene co-expression network construction, as detailed in **Figure 3D**. Using this threshold, a weighted co-expression network was constructed, and filtered genes were grouped into 19 distinct co-expression modules via dynamic tree cutting. Module-trait correlation analysis revealed the black module was significantly correlated with sepsis onset, as well as clinical traits including sepsis etiology and patient age. We further visualized module-phenotype associations with heatmaps and plotted gene significance (GS) against module membership (MM) for the black module. Ultimately, 524 hub genes closely linked to sepsis pathogenesis were screened out from this module, as shown in **Figures 3E, F**.

**Figure 3:**
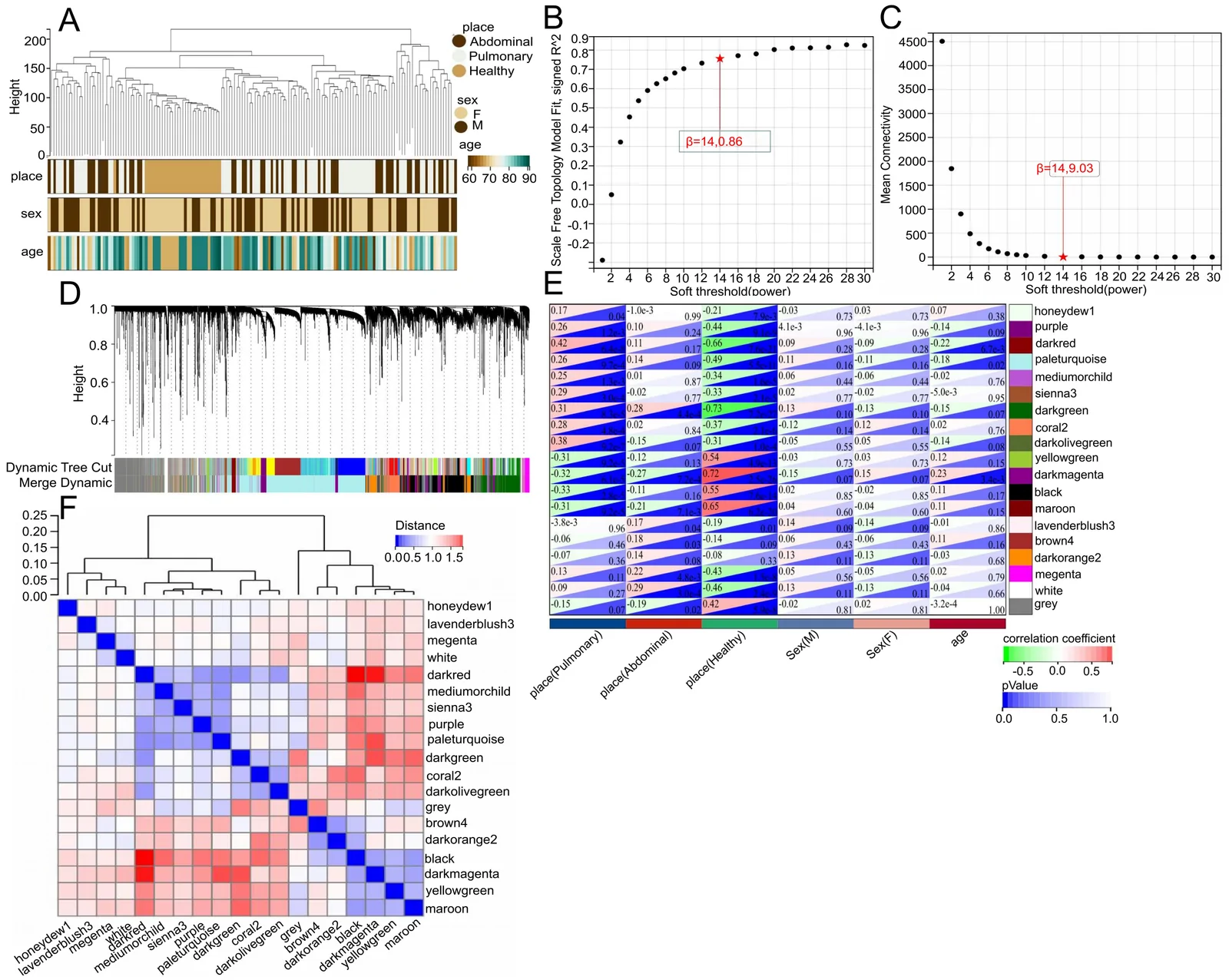
WGCNA analysis of the GSE236713 dataset for sepsis-related gene. **(A)** Cluster dendrogram of clinical variables, including sex, age and sampling site. **(B)** Plot of soft threshold power versus scale-free topology model fit R². The optimal soft threshold β = 14, corresponding to R² = 0.86. **(C)** Mean connectivity curve under varying soft threshold powers. At the optimal β of 14, the mean connectivity reached 9.03. **(D)** Gene clustering dendrogram based on two dynamic tree-cutting methods, with clustering height indicating module partition outcomes. **(E)** Heatmap displaying correlation coefficients and corresponding P values between individual gene co-expression modules and clinical traits (group, sex, age). **(F)** Module eigengene clustering dendrogram; distinct colors denote independent gene co-expression modules. WGCNA, Weighted Gene Co-expression Network Analysis.

### 3.4 Machine learning approaches identify sepsis risk genes

By intersecting above hub genes with DEGs, we identify 15 key genes that influence the pathogenesis of sepsis. There are arylacetamide deacetylase like 3 (*AADACL3*), activin A receptor type 1B (*ACVR1B*), *CHRM1*, interleukin 1 alpha (*IL1A*), insulin receptor (*INSR*), neural precursorcell-expressed developmentally downregulated 8 activating enzyme E1 subunit 1 (*NAE1*), pleckstrin homology like domain family A member 1 (*PHLDA1*), ras and rab interactor 3 (*RIN3*), regulatory associated protein of mTOR complex 1 (*RPTOR*), sialic acid binding Ig like lectin 5 (*SIGLEC5*), solute carrier family 26 member 4 (*SLC26A4*), ubiquitin regulatory X domain protein 6 (UBXN6), protein disulfide isomerase family A member 4 (PDIA4), *PLA2G2A*, and wolf-hirschhorn syndrome candidate 2 (*WHSC2*)(**Figure 4A**). As shown in **Figures 4B, C**, LASSO regression analysis is performed on the 15 co-expressed genes. We utilize their expression values and sample types as continuous predictor variables and categorical responses, respectively (healthy group vs. sepsis group). Through crossover analysis, the genes exhibiting the smallest crossover errors are identified as *AADACL3*, *CHRM1*, *INSR*, *NAE1*, *PLA2G2A*, and *RPTOR*. They are statistically significant (p<0.05) in the logistic regression model (**Figures 4D–F**). Validation is conducted using both linear discriminant analysis (LDA) and quadratic discriminant analysis (QDA) models. The results from the LDA model are summarized as follows: aggregated result: mmce.test.mean = 0.0191, and acc.test.mean = 0.9809 (**Table 2**). In contrast, the QDA model results are as follows: aggregated result: mmce.test.mean = 0.0768417; acc.test.mean = 0.9231583 (**Table 3**). A Gaussian mixture model (GMM) is employed to discriminate among the screened genes. The diagnostic accuracy measured by the area under the curve (AUC) is ranged from 95.58% to 99.97%, as illustrated in **Figure 4G**.

**Figure 4:**
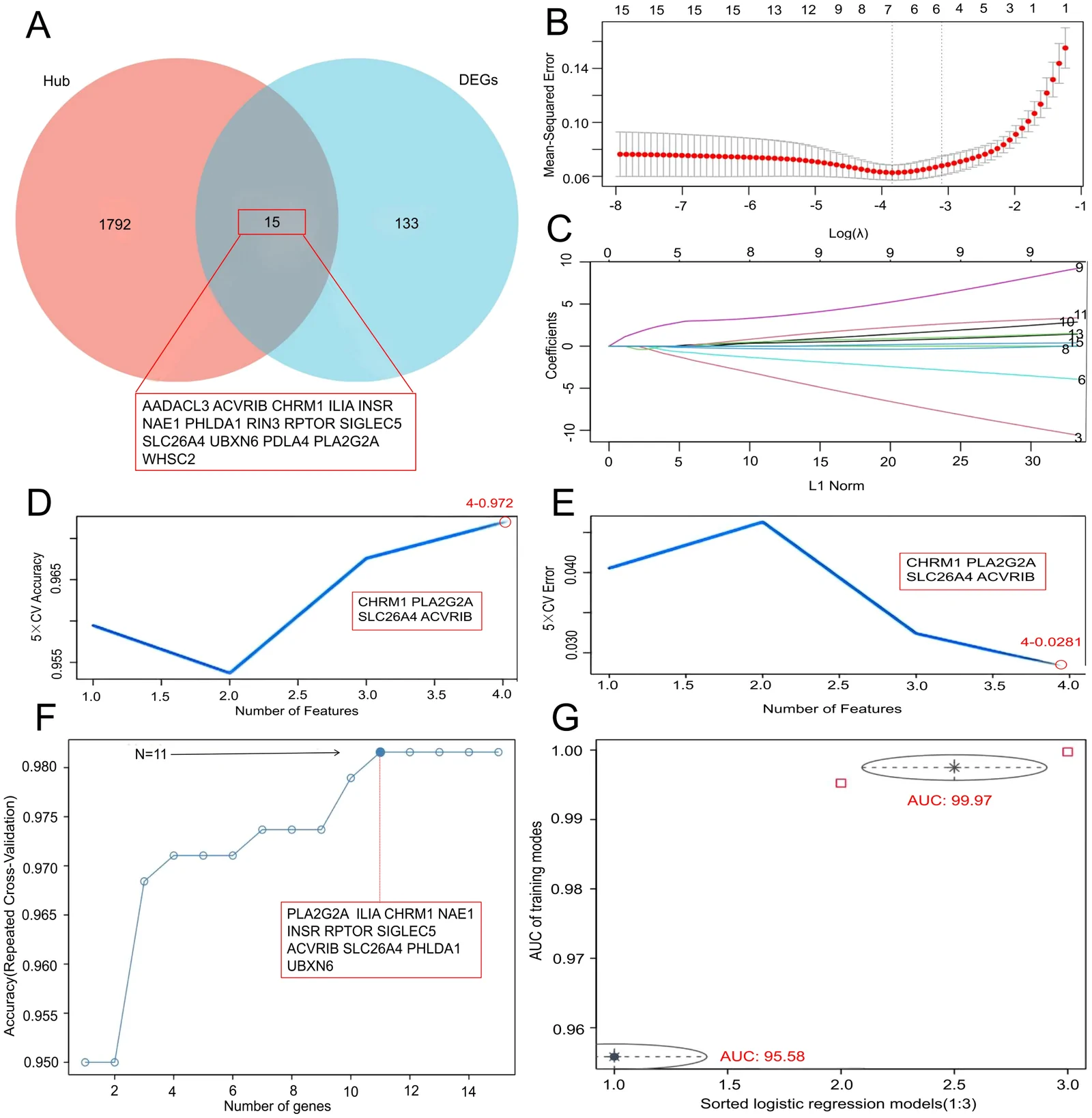
Machine learning-based candidate gene screening and diagnostic model performance assessment. **(A)** Venn diagram showing the overlap of hub genes and DEGs, yielding 15 shared candidate genes. **(B)** LASSO regression for core sepsis gene screening via coefficient shrinkage. **(C)** Trajectory of gene coefficients under L1 regularization in the LASSO regression model. **(D)** Five-fold cross-validation accuracy of the SVM model with varying numbers of candidate feature genes (*CHRM1*, *PLA2G2A*, *SLC26A4*, *ACVR1B*). **(E)** Five-fold cross-validation error of the SVM model as the number of input features increases. **(F)** AUC values of logistic regression models constructed using different gene subsets (11 gene combinations in total). **(G)** AUC distribution of logistic regression models ranked by GMM clustering, with AUC values ranging from 95.58% to 99.97%. All machine-learning classifiers are exploratory and intended only for candidate-gene screening, not for clinical diagnostic application. AUC, Area Under the Curve; LASSO, Least Absolute Shrinkage and Selection Operator; LR, Logistic Regression; SVM, Support Vector Machine; GMM, Gaussian Mixture Model.

**Table 2:** LDA model analysis to verify the accuracy of LASSO regression.

Relative confusion matrix				Absolute confusion matrix			
True	0	1	err	True	0	1	err
0	0.99/0.92	0.01/0.003	0.01	0	1480	20	20
1	0.02/0.08	0.98/0.997	0.02	1	129	6171	129
err	0.08	0.003	0.02	err	129	20	149

**Table 3:** QDA model analysis to verify the accuracy of LASSO regression.

Relative confusion matrix				Absolute confusion matrix			
True	0	1	err	True	0	1	err
0	0.60/1.00	0.40/0.09	0.40	0	902	598	598
1	0.00/0.00	1.00/0.91	0.00	1	0	6300	0
err	0.00	0.09	0.08	err	0	598	598

### 3.5 Analysis of immune infiltration in sepsis

In this study, we conduct a genomic immune infiltration analysis using the leukocyte signature matrix (LM22) dataset. As shown in **Figure 5**, in the memory B cells, plasma cells, naïve CD4+ T cells, monocytes, M2-type macrophages, and eosinophils, the infiltration levels of the patients with sepsis are significantly different from those in the healthy controls (p<0.001). Relative to healthy controls, in the activated naive CD4+ T cells, helper T cells, regulatory T cells, activated natural killer cell (NK) cells, and mast cells, the infiltration levels are significantly different among sepsis patients (p<0.005).

**Figure 5:**
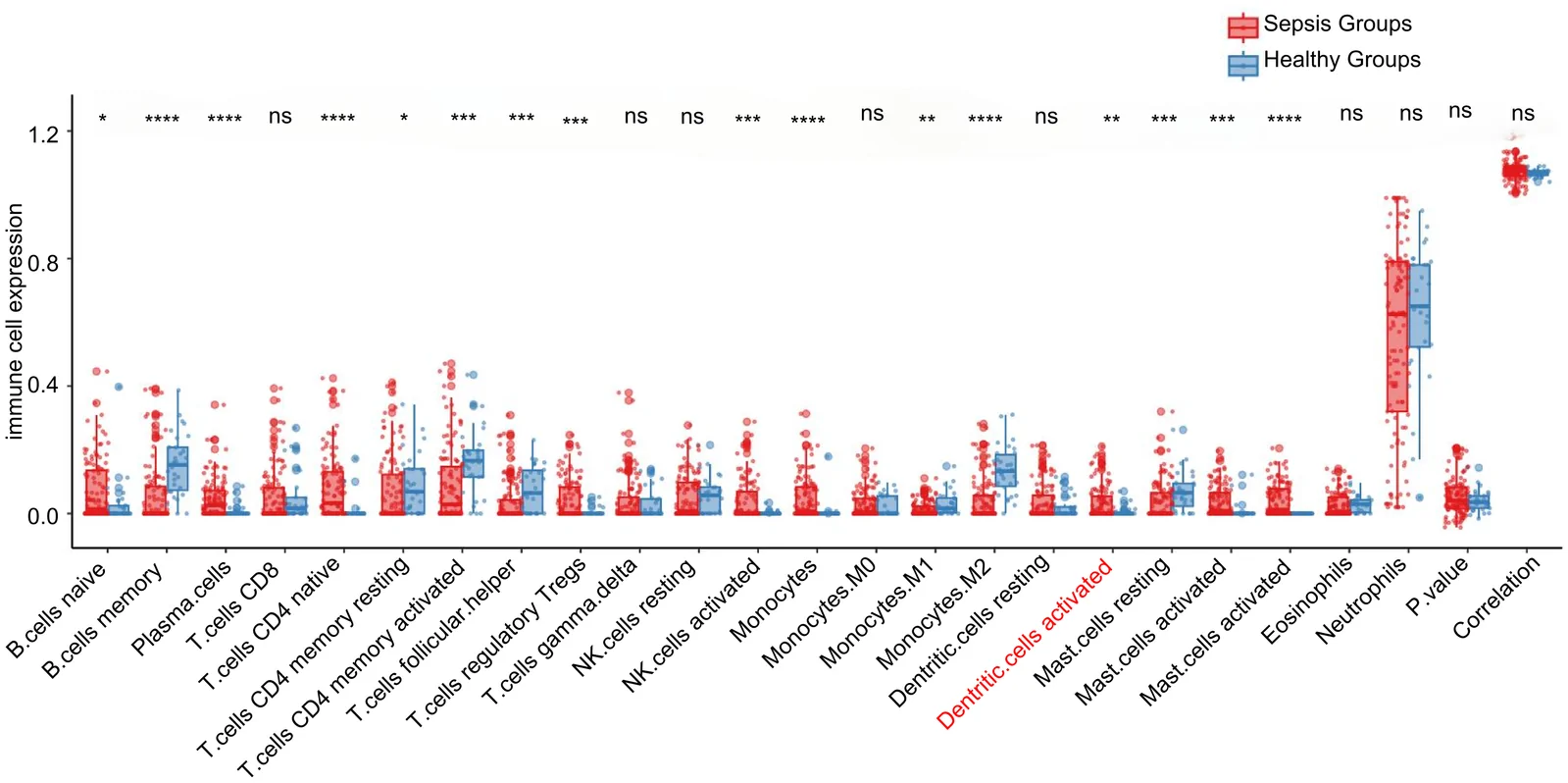
Stacked bar chart illustrating the relative proportions of 22 immune cell subsets in healthy and septic groups. Immune-cell infiltration fractions were estimated by the CIBERSORT algorithm based on the LM22 leukocyte gene-signature matrix. One-way ANOVA was used for group-wise comparisons. ns, not significant; * p<0.05, ** p<0.01, *** p<0.005, **** p<0.001.

Additionally, there is a statistically significant difference in the infiltration of M1-type macrophages and activated dendritic cells between patients with sepsis and healthy controls (p<0.01). Finally, in the naive B cells and resting memory CD4+ T cells, the level of infiltration in patients with sepsis is significantly greater than that in the healthy controls (p<0.05).

### 3.6 Single-gene enrichment analysis

The key genes are identified through LASSO, logistic regression (LR), and support vector machine (SVM) analyses. The result are cross-referenced to yield the final co-expressed genes, which are *CHRM1* and *PLA2G2A* (**Figure 6A**). Single-gene enrichment analysis identifies PLA2G2A as the gene associated with the highest risk of sepsis. GO enrichment analysis reveals that the biological processes involve in interspecies interactions, multicellular organization, immune system responses, localization mechanisms, signaling pathways, disease progression, molecular functions related to localization and binding, and cellular functions pertinent to cellular organisms. Furthermore, KEGG enrichment analysis indicates significant involvement in various metabolic pathways, such as ether lipid metabolism, linoleate metabolism, linolenic acid metabolism, fat digestion and absorption, arachidonic acid metabolism, glycerophospholipid metabolism, pancreatic fluid secretion, vascular smooth muscle contraction, and Ras signaling pathway activities (p<0.05), as shown in **Figures 6B–D**.

**Figure 6:**
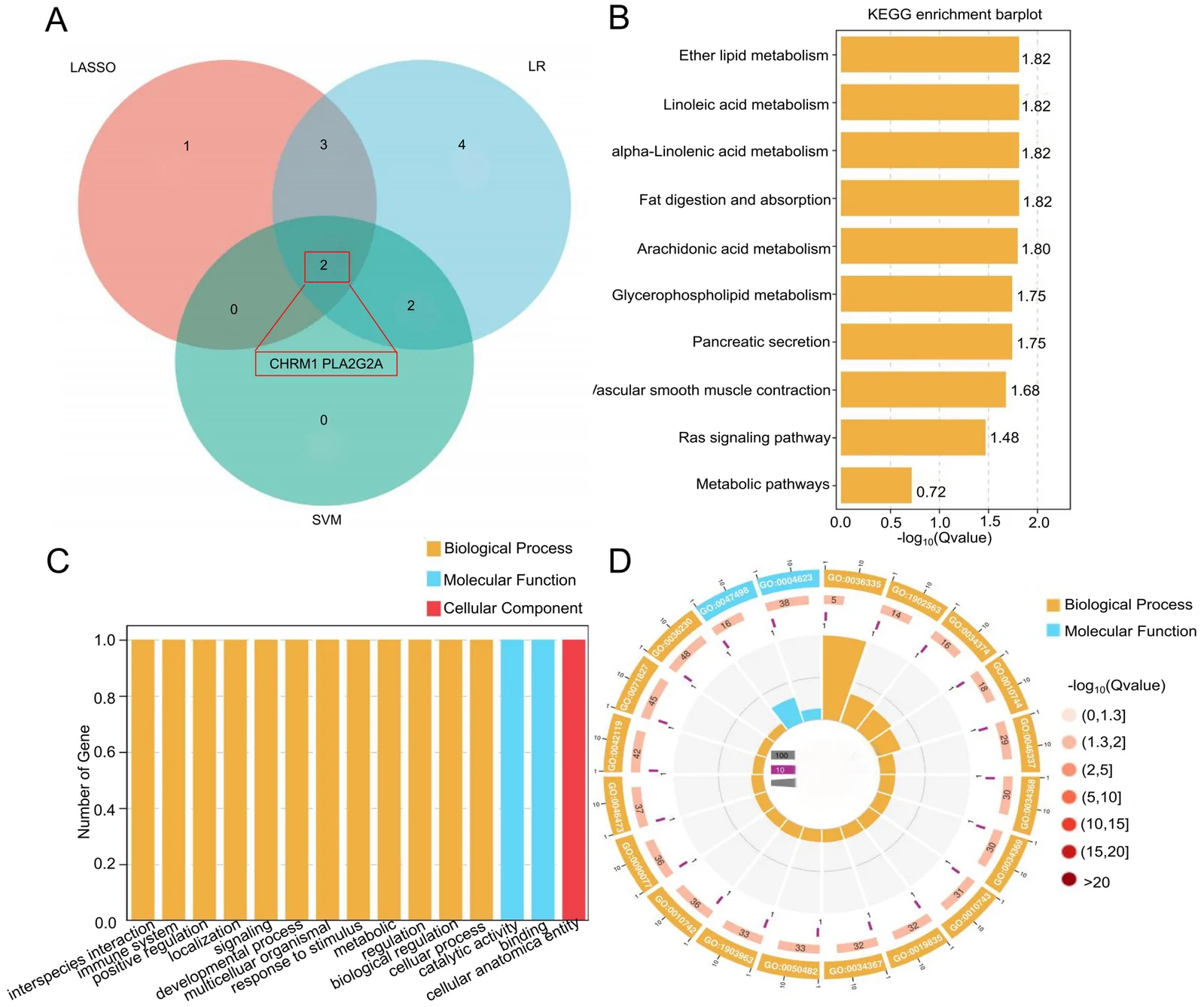
Functional enrichment analysis of two core diagnostic genes (*CHRM1* and *PLA2G2A*). **(A)** Two key candidate genes (*CHRM1* and *PLA2G2A*) identified via LASSO, LR, and SVM algorithms. **(B)** Bar chart displaying significantly enriched KEGG pathways. **(C)** GO enrichment results classified into biological process, cellular component, and molecular function categories; the y-axis corresponds to the count of enriched genes. **(D)** Comprehensive enrichment bubble plot for biological process and molecular function terms. The diagram visualizes gene counts, selected gene numbers, Rich Factor, and −log_10_(Q value) across enrichment terms. GO, Gene Ontology; KEGG, Kyoto Encyclopedia of Genes and Genomes; LR, logistic regression; SVM, support vector machine.

### 3.7 Validation of CLP-induced mouse sepsis model and dynamic serum sPLA2-IIA alterations

CLP-operated mice displayed typical sepsis manifestations including hypoactivity, huddling, anorexia, blunted sensory response and hypothermia, which peaked at 24–48 h and gradually alleviated by 72 h in surviving animals. To biochemically validate model establishment, plasma from sham, CLP-24 h, CLP-48 h and CLP-72 h mice was harvested for ELISA quantification of pro-inflammatory cytokines IL-6, TNF-α and target protein sPLA2-IIA (**Figures 7A–C**). Both IL-6 and TNF-α concentrations were markedly elevated at all CLP time points relative to sham animals (all *p* < 0.001). Circulating sPLA2-IIA was persistently upregulated following CLP, peaking at 24 h and sustaining high levels until 72 h. Correlation analysis demonstrated strong positive linear associations between plasma sPLA2-IIA and IL-6 (*R²* = 0.8652, p < 0.001) as well as TNF-α (*R²* = 0.5088, p = 0.0019), as shown in **Figures 7D, E**. The relationships between plasma sPLA2-IIA and IL-6/TNF-α represent statistical correlation from pooled samples of plasma and serial CLP time points, and explicitly noted that such correlation cannot confirm causal or regulatory effects.

**Figure 7:**
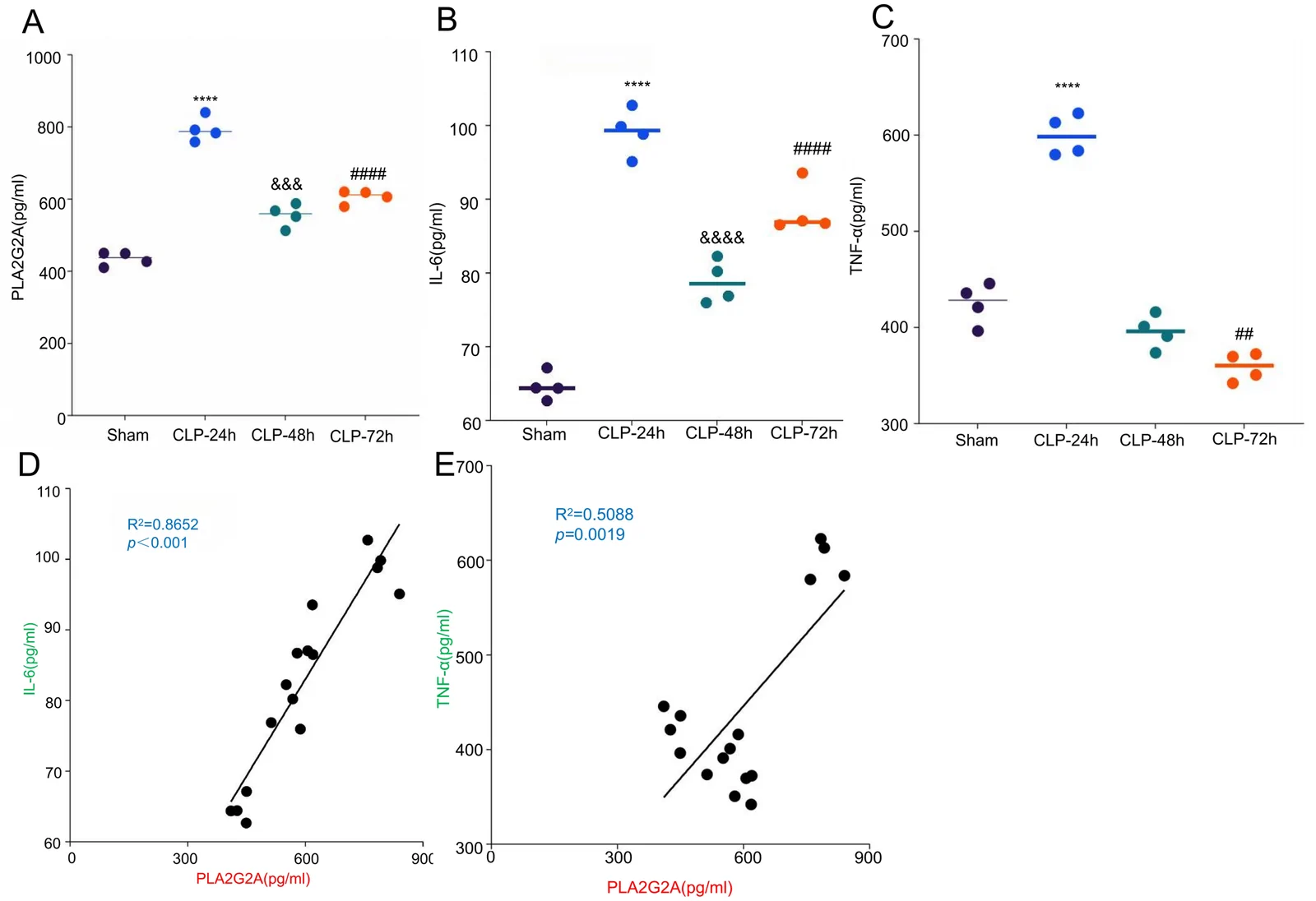
The expression levels of IL-6,TNF-α and were detected by ELISA, and correlation analysis was performed. **(A)** The protein level of sPLA2-IIA (pg/mL) detected by ELISA in the sham group and CLP groups at 24, 48 and 72 h after modeling. **(B)** The protein level of IL-6 (pg/mL) detected by ELISA in the sham group and CLP groups at 24, 48 and 72 h after modeling. **(C)** The protein level of TNF-α (pg/mL) detected by ELISA in the sham group and CLP groups at 24, 48 and 72 h after modeling. **(D)** Correlation analysis between sPLA2-IIA and IL-6. *R^2^* = 0.8652, p < 0.001. **(E)** Linear correlation between sPLA2-IIA and TNF-α (*R^2^* = 0.8652, p < 0.001). CLP, cecum ligation and perforation; IL-6, interleukin 6; TNF-α, tumor necrosis factor α. sPLA2-IIA: phospholipase A2 IIA group; β-actin: internal reference. ** *p* < 0.01; *** *p* < 0.005; **** *p* < 0.001. n = 4 per group.

### 3.8 Tissue-specific sPLA2-IIA expression and multi-organ pathological lesions in CLP sepsis

H&E staining of heart, lung, liver and kidney paraffin sections from sham, CLP-24 h and CLP-72 h groups revealed widespread multi-organ inflammatory injury and inflammatory cell infiltration after CLP, with the most severe pulmonary pathology characterized by alveolar septal thickening, extensive inflammatory infiltration and pulmonary edema (**Figures 8A–L**).

**Figure 8:**
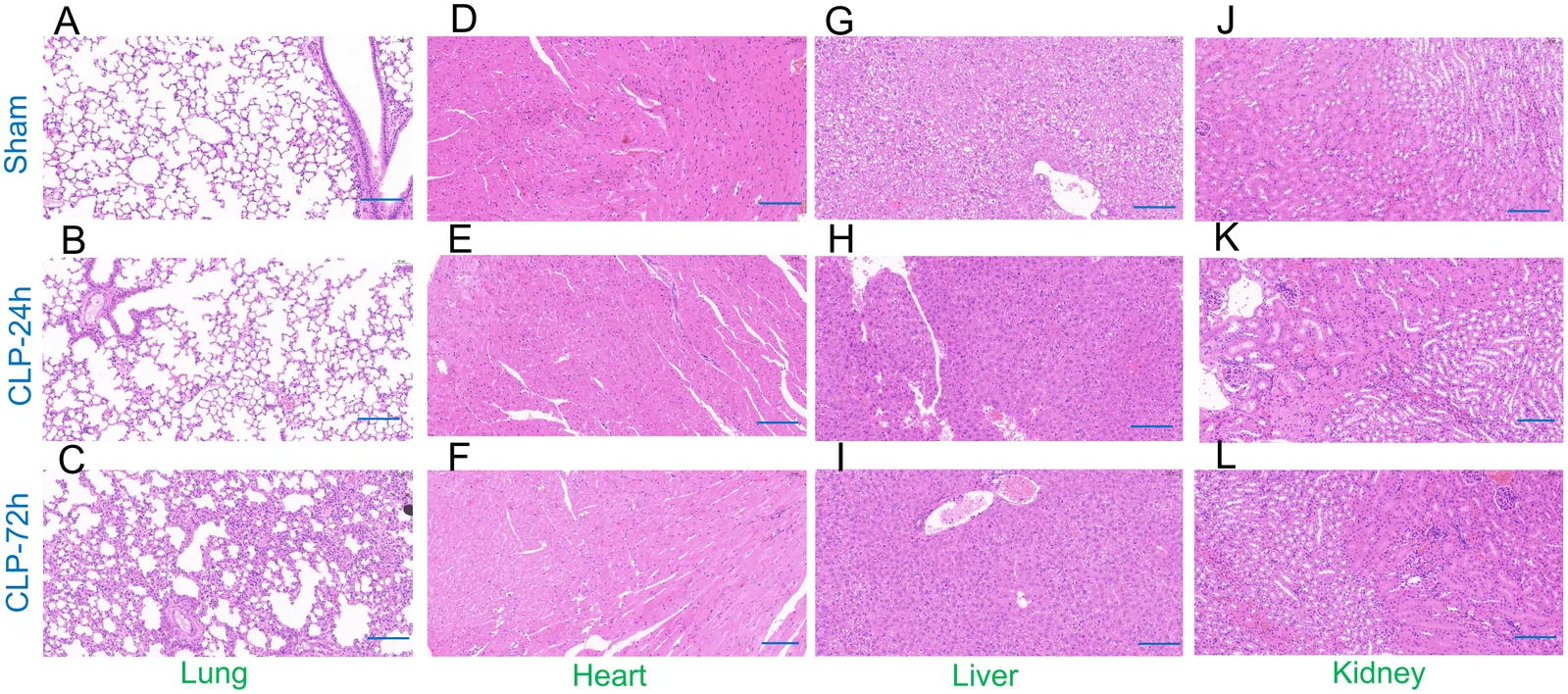
H&E staining of major organs in mice after CLP surgery. **(A)** Lung, sham group: Complete alveolar cavities, thin alveolar septa and rare interstitial inflammatory infiltrates; no evident pulmonary damage. **(B)** Lung, CLP-24 h group: Partially preserved alveolar structure accompanied by inflammatory cell infiltration within alveolar septa. **(C)** Lung, CLP-72 h group: Severe alveolar collapse and irregular alveolar dilatation. **(D)** Heart, sham group: Neatly arranged intact cardiomyocytes with minimal interstitial inflammation; no obvious myocardial lesions. **(E)** Heart, CLP-24 h group: Disordered partial myocardial fibers, interstitial inflammatory infiltration and mild interstitial edema. **(F)** Heart, CLP-72 h group: Massively disorganized cardiomyocytes, widespread interstitial inflammation, marked edema and focal cardiomyocyte degeneration. **(G)** Liver, sham group: Intact hepatic lobular architecture, uniform hepatocytes and negligible interstitial inflammatory infiltration. **(H)** Liver, CLP-24 h group: Moderate hepatocellular swelling and sparse sinusoidal inflammatory cell infiltration. **(I)** Liver, CLP-72 h group: Widespread hepatocellular vacuolar degeneration and dense diffuse sinusoidal inflammatory infiltration. **(J)** Kidney, sham group: Regular renal tubules and intact glomeruli with scarce interstitial inflammation. **(K)** Kidney, CLP-24 h group: Mild swelling of tubular epithelial cells and scattered interstitial inflammatory infiltrates. **(L)** Kidney, CLP-72 h group: Severe tubular vacuolar degeneration, extensive interstitial inflammation and luminal collapse of renal tubules. CLP, cecum ligation perforation group; H&E staining, hematoxylin and eosin staining. n = 6 per group, Scale: 50 µm.

Spleen immunohistochemistry localized sPLA2-IIA-positive cells (**Figures 9A–C**). Virtually no sPLA2-IIA immunoreactivity was detected in sham spleens. At 24 h post-CLP, prominent yellow sPLA2-IIA staining emerged in splenic marginal zone dendritic cells, and diffuse halo-like positive signals were observed at 72 h. Quantitative analysis confirmed that sPLA2-IIA-positive cell density in CLP-24 h spleens increased by 263% compared with sham controls (p < 0.05). Positive signals were predominantly enriched in splenic red pulp macrophages and marginal zone dendritic cells, indicating that these immune cell populations serve as the primary cellular source of sPLA2-IIA during sepsis, as shown in **Figure 9D**. Western blot and immunohistochemistry were applied to profile PLA2G2A protein abundance across major visceral organs (lung, heart, liver, kidney) (**Figure 9E**). sPLA2-IIA protein expression was robustly induced post-CLP; lung, heart and liver exhibited maximal upregulation at 24 h, while kidney sPLA2-IIA peaked at 48 h.

**Figure 9:**
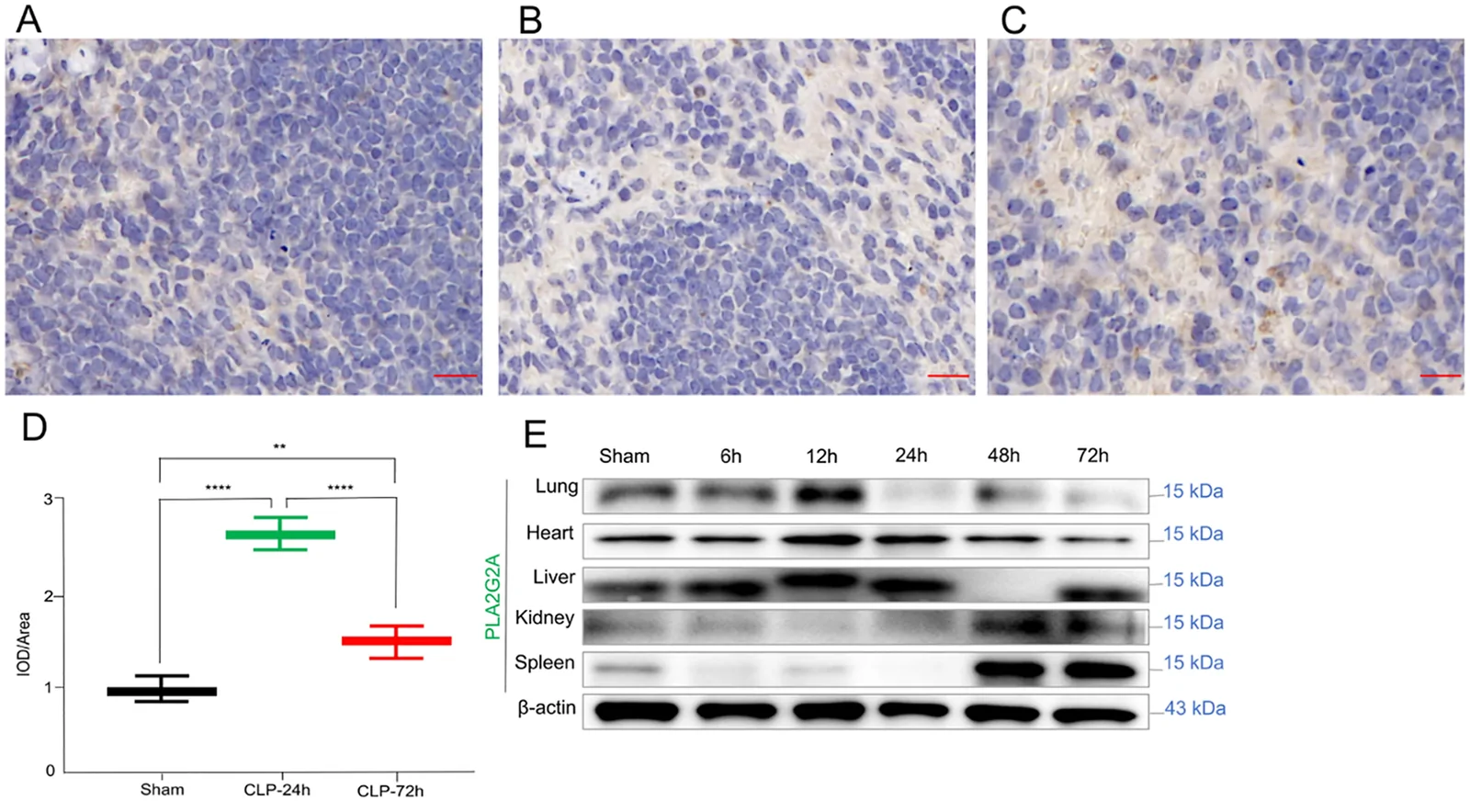
Detection of sPLA2-IIA expression in multiple mouse organs after CLP surgery. **(A)** Immunohistochemical staining of the mouse spleen after CLP. Splenic DCs display extremely weak PLA2G2A immunoreactivity, with barely detectable positive staining signals in sham group. **(B)** Moderate sPLA2-IIA positive staining is observed in splenic DCs in CLP-24 h group. **(C)** Splenic DCs exhibit abundant and intense sPLA2-IIA staining in CLP-72 h groups. **(D)** Quantitative analysis of sPLA2-IIA immunoreactivity calculated by IOD/Area. **(E)** Western blot analysis of sPLA2-IIA (15 kDa) at sequential time points, with β-actin (43 kDa) as reference. IOD/area, average optical density analysis. Statistical analysis of the protein gray values was performed using one-way ANOVA with two-by-two comparisons, ** p<0.01; **** p<0.001. CLP, Cecum Ligation Perforation. n = 6 per group, Scale: 20 µm.

### 3.9 *PLA2G2A* mRNA expression following CLP

To address the biological plausibility of sPLA2-IIA protein detection in C57BL/6J mice, we performed RT-qPCR to quantify *PLA2G2A* mRNA expression in relevant tissues and peripheral blood cells from sham- and CLP-operated mice. As shown in **Supplementary Figure S2**, basal *PLA2G2A* transcript levels were low under sham conditions, consistent with the known *PLA2G2A* frameshift mutation in the C57BL/6J substrain. As shown in **Figure 10**, following CLP-induced polymicrobial sepsis, *PLA2G2A* mRNA was significantly upregulated in the lung, kidney, liver, and peripheral blood cells compared with sham controls (all p < 0.05). Importantly, because C57BL/6J mice carry a frameshift mutation in *PLA2G2A*, the induced transcripts encode a truncated, non-functional protein variant. Therefore, the elevated mRNA levels observed after CLP reflect transcriptional upregulation of the mutant allele rather than production of intact, catalytically competent wild-type sPLA2-IIA.

**Figure 10:**
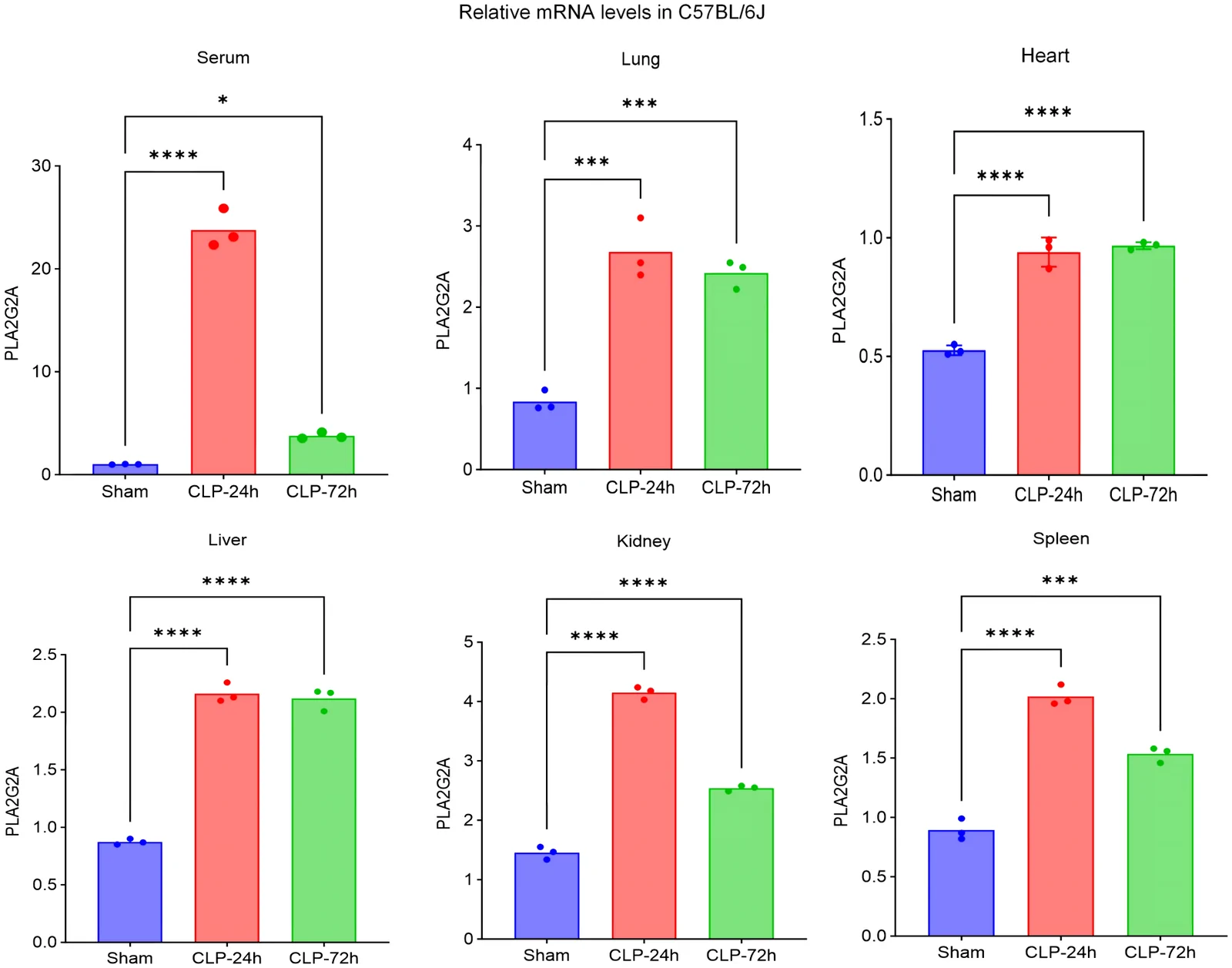
Dynamic changes of Pla2g2a mRNA in serum and multiple organs after CLP-induced polymicrobial sepsis in C57BL/6J mice. qPCR analysis of Pla2g2a relative mRNA expression in serum, lung, heart, liver, kidney and spleen. Mice were subjected to sham operation or CLP surgery and sacrificed at 24 h and 72 h post-procedure. Each dot denotes one independent biological replicate (individual mouse). Data represent mean ± SD. One-way ANOVA and Tukey’s multiple comparison test. P < 0.05, **P < 0.001, ***P < 0.0001. CLP, Cecum Ligation Perforation. n = 3 per group.

## 4 Discussion

In the present study, we integrated human transcriptomic data from public databases with multiple machine-learning algorithms to screen sepsis-associated candidate biomarkers, and further performed in-vivo experimental validation using cecum ligation and perforation (CLP)-induced murine sepsis models. We identified *PLA2G2A* as a promising candidate biomarker for sepsis in mice. Our in-vivo data demonstrated markedly elevated sPLA2-IIA levels in circulation and multiple visceral organs during CLP-triggered sepsis. Plasma sPLA2-IIA concentrations were positively correlated with pro-inflammatory cytokines IL-6 and TNF-α. Splenic macrophages and dendritic cells were further recognized as the predominant cellular sources of sPLA2-IIA under septic conditions.

Previous clinical studies have validated circulating sPLA2-IIA (encoded by PLA2G2A) as a diagnostic biomarker for human sepsis (16, 17). Nevertheless, human-based investigations are largely restricted to peripheral serum measurements, and direct evidence regarding in-vivo tissue distribution and the cellular origin of PLA2G2A during sepsis remains scarce, since human immune-organ specimens are difficult to obtain. In the present work, we performed unbiased candidate-gene screening integrating transcriptomic datasets and multiple machine-learning algorithms, followed by in-vivo validation in CLP-induced murine sepsis models. Beyond confirming elevated circulating and tissue-level PLA2G2A together with its correlation with pro-inflammatory cytokines, we further identified splenic macrophages and dendritic cells as the predominant cellular sources of PLA2G2A under septic conditions. Our study extends previous clinical observations from circulating biomarker detection toward tissue- and cellular-level in-vivo evidence, laying pre-clinical groundwork for future neutralizing-antibody or inhibitor interventions as well as subsequent clinical translation.

Using the GSE236713 dataset from the GEO database under strict filtering criteria, we screened 158 co-expressed differentially expressed genes (DEGs) related to sepsis. These DEGs are involved in cellular immune responses and the regulation of inflammatory processes. Functional enrichment analyses indicated that these genes are predominantly enriched in metabolic and immune-activation pathways reported in previous sepsis cohorts (21). KEGG enrichment further highlighted several immune-metabolic cascades, including the AMPK, PI3K-Akt, and mTOR signaling pathways; dysregulation of these pathways is well-documented in sepsis-related immune dysfunction (22, 23). By combining WGCNA and multiple machine-learning approaches including LASSO, LDA, QDA, SVM and GMM, six hub genes (*AADACL3*, *CHRM1*, *INSR*, *NAE1*, *PLA2G2A*, and *RPTOR*) were prioritized. Each of these genes has been implicated in cellular stress adaptation, tumor biology, metabolic homeostasis or autophagy in earlier studies (24–31). Among them, *PLA2G2A* was ultimately as the candidate for sepsis-relevant research. Weighted gene co-expression network analysis constructed scale-free gene co-expression modules and uncovered that the black module was strongly correlated with sepsis pathogenesis, as well as clinical traits including sepsis etiology, patient age and sex. Hub genes within this module may offer potential targets for precision-oriented sepsis research. We further validated our bioinformatic findings using the classic CLP mouse sepsis model (32). Consistent with clinical observations in human sepsis patients, CLP-challenged mice exhibited robust elevation of serum IL-6 and TNF-α alongside widespread multi-organ pathological damage. Histological examinations revealed typical septic injuries across major organs: pulmonary hemorrhage and alveolar destruction, myocardial fiber disorganization, hepatocyte swelling and inflammatory infiltration, and severe tubular damage in the kidney (33–37). After CLP-induced sepsis establishment, sPLA2-IIA protein abundance was upregulated across multiple visceral organs. Increased cardiac sPLA2-IIA may contribute to sepsis-provoked myocardial injury, in line with prior work describing sPLA2-IIA-mediated sympathetic over-activation in cardiac pathological states (38). Elevated pulmonary sPLA2-IIA, meanwhile, may drive local inflammatory and fibrotic signaling within lung tissue (18). Immunohistochemistry localized sPLA2-IIA-positive signals primarily to splenic macrophages and dendritic cells in the splenic red pulp and marginal zone. sPLA2-IIA secreted by these immune cells may participate in modulating macrophage phagocytosis, inflammatory mediator secretion, as well as dendritic-cell-mediated antigen presentation and immune activation during different phases of sepsis.

## 5 Limitations and future perspectives

This study has several important limitations. First, human transcriptomic analysis relied solely on the GSE236713 public dataset containing longitudinal repeated samples from individual patients. Machine-learning classifiers were evaluated by sample-level five-fold cross-validation and have not been validated in a fully independent external cohort. Therefore, the reported high diagnostic AUC values should be interpreted as purely exploratory rather than clinically applicable. Second, comparisons between sepsis patients and healthy controls cannot establish sepsis-specificity for PLA2G2A/sPLA2-IIA; elevated sPLA2-IIA may represent a marker of general systemic inflammation. Third, our in-vivo CLP mouse experiments only demonstrate correlative associations among sPLA2-IIA abundance, pro-inflammatory mediators and disease severity. Further loss-of-function and gain-of-function experiments are required to establish direct causal roles. Fourth, C57BL/6J mice carry a well-documented frameshift mutation within P*LA2G2A* (19). Detected protein signals correspond to truncated mutant peptide rather than catalytically-competent wild-type sPLA2-IIA. Still, immunoreactive truncated sPLA2-IIA has been reported in inflamed C57BL/6J lung tissue under severe inflammatory stress (20). Follow-up work using C57BL/6N or genetically complemented mouse models will be necessary to dissect wild-type protein function. Finally, all animal experiments utilised only male mice; whether these findings can be generalised to female animals remains to be determined. Regarding clinical translation, substantial practical challenges remain, including variable baseline circulating sPLA2-IIA concentrations, confounding inflammation from chronic comorbidities, and the absence of standardised detection protocols and clinical cut-off values.

## 6 Conclusion

Based on the GSE236713 dataset, we conducted comprehensive bioinformatics analyses, including DEG screening, GO/KEGG enrichment analysis and WGCNA. Combined with multiple machine learning algorithms (LASSO, LR, GMM and SVM), key sepsis-associated genes were screened, with PLA2G2A selected for experimental validation. A stable murine sepsis model was established in C57BL/6J mice, which exhibited increased IL-6 and TNF-α levels as well as multi-organ damage. *In vivo* experiments confirmed that sPLA2-IIA was markedly upregulated in septic tissues and positively correlated with proinflammatory factors. Collectively, this study provides supplementary experimental evidence supporting the reported association between *PLA2G2A* and sepsis through machine learning-based bioinformatics analysis and experimental verification. Our results provide a promising diagnostic target and offer valuable insights for future research on sepsis pathogenesis.

## Data Availability

The original contributions presented in the study are included in the article/**Supplementary Material**. Further inquiries can be directed to the corresponding authors.

## Glossary

AADACL3: Arylacetamide deacetylase like 3
ACVR1B: Activin A Receptor Type 1B
AMPK: Adenosine 5’-monophosphate (AMP)-activated protein kinase
AUC: Area under the curve
CHRM1: Cholinergic receptor muscarinic 1
CLP: Cecum ligation and perforation
DEGs: Differentially expressed genes
GEO: Gene expression omnibus
GMM: Gaussian mixture model
GS-MM: Gene significance vs. module membership
HRP: Horseradish Peroxidase
IHC: Immunohistochemistry
IL1A: Interleukin 1 alpha
IL-6: Interleukin-6
INSR: Insulin receptor
KEGG: Kyoto encyclopedia of genes and genomes
LASSO: Least absolute shrinkage and selection operator
LDA: Linear discriminant analysis
LM22: Leukocyte signature matrix
LR: Logistic regression
MAD: Median absolute deviation
mTOR: Mechanistic target of rapamycin
NAE1: NEDD8 activating enzyme E1 subunit 1
INSR: Insulin receptor
NAE1: NEDD8 activating enzyme E1 subunit 1
NK: Natural killer cell
PDIA4: Protein disulfide isomerase family A member 4
PHLDA1: Pleckstrin homology like domain family A member 1
PI3K-Akt: Phosphatidylinositol 3-Kinase/Protein Kinase B
PLA2G2A: Secreted phospholipase A2
PVDF: Polyvinylidene Fluoride
PDIA4: Protein disulfide isomerase family A member 4
QDA: Quadratic discriminant analysis
RIN3: Ras and rab interactor 3
RPTOR: Regulatory associated protein of mTOR complex 1
SIGLEC5: Sialic acid binding Ig like lectin 5
SLC26A4: Solute carrier family 26 member 4
SVM: Support vector machine
UBXN6: Ubiquitin regulatory X domain protein 6
WB: Western blotting
WGCNA: Weighted gene co-expression network analysis
WHSC2: Wolf-hirschhorn syndrome candidate 2
