# Supplementary material for: PLA2G2A is a candidate biomarker for sepsis diagnosis in mice through machine learning algorithms
Source: Front Immunol. 2026 Sep 30;17:1937718. doi: 10.3389/fimmu.2026.1937718 (PMC13661449; doi:10.3389/fimmu.2026.1937718)
Supplement: Supplementary file 1 [file Table1.docx]

Table S1. Key resources

| REAGENTS or ANTIBODIES | SOURCE | IDENTIFIER | COUNTRY |
| --- | --- | --- | --- |
| Isoflurane | RWD | R510-22-10 | China |
| NaCl | Servicebio | G4702 | China |
| Phosphate buffered saline | Beyotime | C0221A | China |
| RIPA lysis buffer | Beyotime | P0013B | China |
| [Mouse IL6 ELISA Kit](https://boster.com/index/products/productsDetail?goods_sn=EK0411) | BOSTER | EK0411 | China |
| Mouse TNF Alpha ELISA Kit | BOSTER | EK0527 | China |
| Paraformaldehyde solution | Beyotime | P0099 | China |
| 10% formalin | Sigma-Aldrich | F5554, | USA |
| Xylene substitute | Beyotime | 8052-41-3 | China |
| Ethanol solutions | WANVI | W617032 | China |
| Tris-EDTA Antigen Retrieval Solution | Beyotime | P0084 | China |
| Rabbit serum | Boster Biological Technology | AR1010 | China |
| PLA2G2A | Affinity | DF6366 | China |
| Anti-rabbit IgG, HRP-linked Antibody | Cell Signaling Technology | 7074 | USA |
| Diaminobenzidine Chromogenic solution | Sigma-Aldrich | D12384 | USA |
| Loading buffer | Thermo-Scientific | 26616 | USA |
| Omni-Easy^TM^ Gel Rapid Preparation Kit | Epizyme Biomedical Technology | PG212 | China |
| Blocking solution | NcmBlot | P30500 | China |
| PLA2G2A antibody | Affinity | DF6366 | China |
| β-Actin monoclonal antibody | Proteintech | 66009-1-Ig | China |
